# Statins associated with improved heart structure and function

**Published:** 2017

*Statins are associated with improved heart structure andfunction, according to* research presented recently atEuroCMR 2017. The benefits were above and beyond thecholesterol-lowering effect of statins.

‘Statins are primarily used to lower cholesterol’, said leadauthor Dr Nay Aung, a cardiologist and Wellcome Trustresearch fellow, William Harvey Research Institute, QueenMary University of London, UK. ‘They are highly effectivein preventing cardiovascular events in patients who have hada heart attack or are at risk of heart disease.’

He continued: ‘Statins have other beneficial, non-cholesterollowering,effects. They can improve the function of the bloodvessels, reduce inflammation, and stabilise fatty plaques in theblood vessels. Studies in mice and small studies in humanshave shown that statins also reduce the thickness of heartmuscle but this needed to be confirmed in a larger study.’

This study investigated the association between statinsand heart structure and function. The study included 4 622people without cardiovascular disease from the UK Biobank,a large community-based cohort study. Cardiac magneticresonance imaging was used to measure left and rightventricular volumes and left ventricular mass. Informationon statin use was obtained from medical records and a selfreporting questionnaire.

The relationship between statin use and heart structure and function was assessed using a statistical technique called multiple regression, which adjusts for potential confounders that can have an effect on the heart, such as ethnicity, gender, age, and body mass index (BMI).

Nearly 17% of participants were taking statins. Those taking statins were older, had higher BMI and blood pressure, and were more likely to have diabetes and hypertension. ‘This was not surprising because we prescribe statins to patients at high risk of heart disease and these are all known risk factors’, said Dr Aung.

Patients taking statins had a 2.4% lower left ventricular mass and lower left and right ventricular volumes. Dr Aung said: ‘People using statins were less likely to have a thickened heart muscle (left ventricular hypertrophy) and less likely to have a large heart chamber. Having a thick, large heart is a strong predictor of future heart attack, heart failure or stroke and taking statins appears to reverse the negative changes in the heart which, in turn, could lower the risk of adverse outcomes.’

‘It is important to note that in our study, the people taking statins were at higher risk of having heart problems than those not using statins yet they still had positive heart remodelling compared to the healthier control group’, added Dr Aung.

In terms of how statins might reduce the thickness and volume of the heart, Dr Aung said several studies have demonstrated that statins reduce oxidative stress and dampen the production of growth factors that stimulate cell growth. Statins also increase the production of nitric oxide by the cells lining the blood vessels, leading to vasodilatation, improved blood flow, lower blood pressure, and lower stress on the heart, which is less likely to become hypertrophied.

The findings raise the issue of extending statin prescriptions to anyone above the age of 40 years, but Dr Aung said that was probably not the way to go.

‘There are clear guidelines on who should receive statins’, he said. ‘There is debate about whether we should lower the bar and the question is when do you stop. What we found is that for patients already taking statins, there are beneficial effects beyond cholesterol lowering and that’s a good thing. But instead of a blanket prescription, we need to identify people most likely to benefit, that is, personalised medicine.’

Dr Aung said: ‘A dual approach should be considered to identify people who will benefit most from statins. That means looking at not only clinical risk factors, such as smoking and high blood pressure, but also genetic (hereditary) factors, which can predict individuals’ response to statins. This is an area of growing interest and one that we are also investigating in our lab with our collaborators.’
